# Genotype distribution of *Pneumocystis jirovecii* in immunocompromised patients: a single-center molecular epidemiology study

**DOI:** 10.3389/ffunb.2026.1841693

**Published:** 2026-05-20

**Authors:** Cecília Salete Alencar, Marcello Mihailenko Chaves Magri, Helio Hehl Caiaffa Filho, Ester Cerdeira Sabino

**Affiliations:** 1 Divisão de Laboratório Central, Departamento de Patologia, Hospital das Clínicas da Faculdade de Medicina da Universidade de São Paulo, São Paulo, Brazil; 2 Divisão de Clínica de Moléstias Infecciosas e Parasitárias, Hospital das Clinicas da Faculdade de Medicina da Universidade de São Paulo, São Paulo, Brazil

**Keywords:** genotyping, immunocompromised hosts, mixed infections, molecular epidemiology, *Pneumocystis jirovecii*

## Abstract

**Background:**

*Pneumocystis jirovecii* pneumonia (PCP) remains a major opportunistic infection affecting both HIV-infected and non-HIV immunocompromised individuals. Key aspects of its transmission dynamics in complex healthcare environments remain poorly understood.

**Methods:**

We conducted a descriptive molecular epidemiology study to characterize the genetic diversity of *P. jirovecii* in a large tertiary-care hospital in São Paulo, Brazil. Respiratory samples collected from hospitalized and outpatient individuals undergoing molecular investigation for PCP between 2014 and 2015 were analyzed. PCR-positive specimens were genotyped by sequencing the mitochondrial large subunit rRNA (mtLSUrRNA) locus.

**Results:**

Among 80 patients tested, 58 samples were successfully sequenced, including individuals living with HIV/AIDS (39.7%), solid organ transplant recipients (32.8%), and patients with other immunosuppressive conditions (27.6%). Three genotypes were identified. Genotype 3 predominated across all clinical groups (69%), whereas genotypes 2 and 1 accounted for 12% and 7%, respectively. Mixed infections were detected in 12% of cases.

**Conclusions:**

In this descriptive molecular epidemiology study, genotype 3 predominated across diverse immunocompromised populations within a large tertiary-care hospital. These findings should be interpreted as hypothesis-generating and may reflect underlying genotype distribution or shared environmental exposure. Further studies incorporating higher-resolution typing methods and clinical correlation are needed to better define transmission pathways.

## Introduction

*Pneumocystis jirovecii* pneumonia (PCP) remains a major opportunistic infection affecting HIV-infected and non-HIV immunocompromised individuals worldwide (Wills et al., 2021; Alsayed et al., 2022; Asai et al., 2022; Cheng et al., 2024; Denning, 2024; Saadatzadeh et al., 2024). Although its incidence has declined among people living with HIV following antiretroviral therapy, PCP continues to cause substantial morbidity and mortality among transplant recipients, patients with malignancies, and individuals receiving prolonged immunosuppressive therapies (Wills et al., 2021; Alsayed et al., 2022; Asai et al., 2022; Cheng et al., 2024; Saadatzadeh et al., 2024). Several aspects of *P. jirovecii* epidemiology remain poorly understood (Vera and Rueda, 2021). Evidence of person-to-person airborne transmission derives from nosocomial clusters and genotypic similarities among epidemiologically linked cases (Gupta et al., 2011; Yiannakis and Boswell, 2016), yet the relative contribution of recent acquisition versus reactivation remains debated, underscoring the need for molecular epidemiological investigations to clarify transmission pathways and support infection control strategies in high-risk healthcare settings (Gupta et al., 2009; Gupta et al., 2011; Pasic et al., 2020). PCR-based tools enable characterization of genetic diversity through mitochondrial and nuclear loci, including mtLSUrRNA, cytochrome b, DHFR, DHPS, SOD, β-tubulin, 26S rRNA, and ITS regions (Beard et al., 2000; Gupta et al., 2009; Gupta et al., 2011; Pasic et al., 2020). The mtLSUrRNA locus is a stable marker for genotypic characterization and detection of mixed infections (Beard et al., 2000). Because genotype distribution varies geographically and remains insufficiently characterized in many middle-income countries, we investigated the genetic diversity of P*. jirovecii* among immunocompromised populations attending a large tertiary-care hospital complex in São Paulo, Brazil, to explore epidemiological patterns and generate hypotheses regarding potential transmission dynamics.

## Materials and methods

This descriptive molecular epidemiology study included respiratory samples collected between April 2014 and July 2015 from hospitalized and outpatient individuals undergoing molecular investigation for suspected Pneumocystis jirovecii pneumonia (PCP) at the Hospital das Clínicas da Faculdade de Medicina da Universidade de São Paulo, a large tertiary-care hospital complex comprising five medical institutes and approximately 2,000 beds. Each sample corresponded to a single patient. A total of 80 consecutive respiratory samples were analyzed, and 58 PCR-positive specimens were included in the molecular genotyping analysis.

Genotyping was performed by sequencing the mitochondrial large subunit rRNA (mtLSUrRNA) region, selected for its genetic stability and established use in PCP epidemiological studies (Gupta et al., 2009). DNA was extracted from 200 µL of respiratory samples using the QIAamp DNA Blood Mini Kit (QIAGEN, Hilden, Germany). A ~370 bp mtLSUrRNA fragment was amplified using primers pAZ102-E (5′-GATGGCTGTTTCCAAGCCCA-3′) and pAZ102-H (5′-GTGTACGTTGCAAAGTACTC-3′) (Beard et al., 2000). PCR reactions (50 µL) contained 0.4 mM dNTPs, 2.5 mM MgCl_2_, 0.2 pmol/µL of each primer, and 1.5 U Taq DNA polymerase, followed by initial denaturation at 94 °C for 1 min, 35 cycles of 94 °C for 45 s, 55 °C for 45 s, and 72 °C for 2 min, and final extension at 72 °C for 10 min.

Amplicons were purified with the QIAquick PCR Purification Kit (QIAGEN) and sequenced using the BigDye Terminator Cycle Sequencing Kit on an ABI PRISM 3130 instrument (Applied Biosystems, Foster City, CA). Sequencing employed the same primers (Wakefield et al., 1990), chromatograms were analyzed with SEQUENCHER software (Gene Codes Corporation, Ann Arbor, MI). Genotypes were defined based on polymorphisms at nucleotide positions 85 and 248 of the mtLSUrRNA gene, as described by Beard et al. (2000), where genotype 1 corresponds to 85C/248C, genotype 2 to 85A/248C, and genotype 3 to 85T/248C (Beard et al., 2000).

## Results

The 80 samples included in the molecular study corresponded to 80 individual patients with a mean age of 47 years (range 1–79), of whom 60% (48/80) were male. Thirty-one patients (39%) had HIV/AIDS, 26 (33%) were solid organ transplant recipients, and 23 (29%) had other underlying conditions. Of the 80 respiratory samples analyzed, 58 yielded sequences suitable for genotyping. Among the 58 successfully sequenced samples, 23 (39.7%) were from HIV/AIDS patients, 19 (32.8%) from transplant recipients, and 16 (27.6%) from patients with other comorbidities. Patients were treated across different institutes within the HCFMUSP hospital complex.

**Figure 1:**
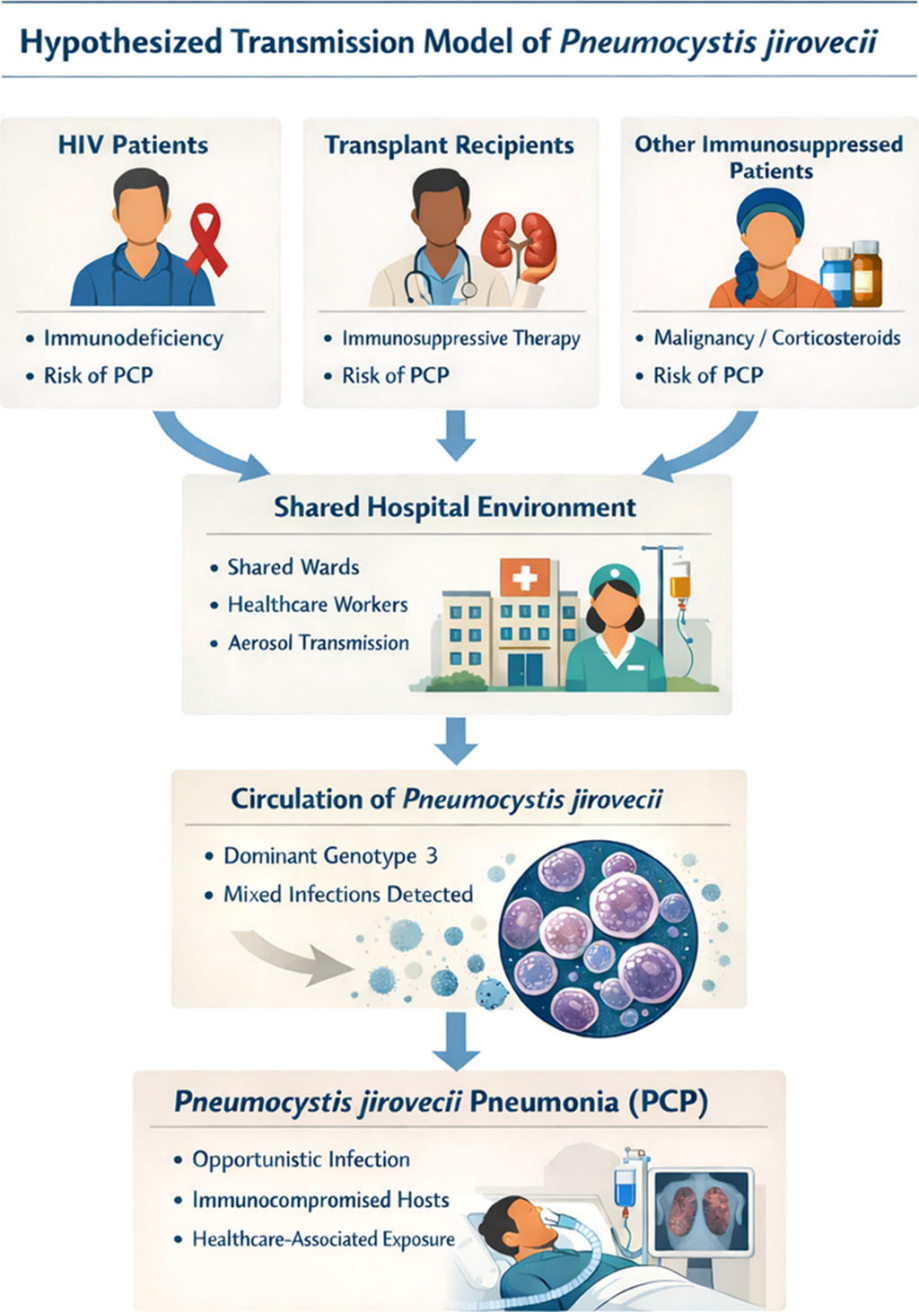
Hypothesized transmission model of *Pneumocystis jirovecii* in a tertiary-care hospital complex. The concept of a “shared hospital environment” is presented as a hypothetical framework, as no spatial or temporal overlap data were available for the included patients. Immunocompromised populations, including patients with HIV infection, solid organ transplant recipients, and other immunosuppressed individuals (e.g., malignancy or corticosteroid therapy), may share hospital environments where airborne transmission of *P. jirovecii* could occur. The circulation of the organism within these settings may result in a predominance of specific genotypes, as observed in this study with genotype 3, as well as the occurrence of mixed infections. In susceptible hosts, exposure to circulating strains may lead to the development of PCP, an opportunistic infection affecting immunocompromised patients. This figure represents a hypothetical conceptual framework illustrating potential pathways of *P. jirovecii* circulation and should not be interpreted as direct evidence of transmission.

**Table 1** summarizes the mtLSUrRNA genotype distribution. Genotypes were defined by single nucleotide polymorphisms at positions 85 and 248. Three genotypes were identified among the 58 samples: genotype 3 (85T/248C) predominated (69%, 40/58), followed by genotype 2 (12%, 7/58) and genotype 1 (7%, 4/58). Mixed infections involving two genotypes were detected in seven cases (12%). Genotype 3 predominated across all clinical groups, and no mixed infections were observed among solid organ transplant recipients. To summarize the potential epidemiological pathways suggested by our findings, a conceptual model of Pneumocystis jirovecii circulation in the hospital environment is presented in **Figure 1**.

**Table 1:** Distribution of *Pneumocystis jirovecii* genotypes (mtLSUrRNA) by clinical group.

Patient group	Total samples	Genotype 1 (85C/248C)	Genotype 2 (85A/248C)	Genotype 3 (85T/248C)	Mixed infections (total)	Mixed subtypes
HIV/AIDS	23	2 (8.7%)	4 (17.4%)	13 (56.5%)	4 (17.4%)	1 + 2 (1), 1 + 3 (1), 2 + 3 (2)
Solid organ transplant	19	1 (5.3%)	2 (10.5%)	16 (84.2%)	0 (0.0%)	–
Other comorbidities	16	1 (6.2%)	1 (6.2%)	11 (68.8%)	3 (18.8%)	1 + 3 (3)
Total	58	4 (6.9%)	7 (12.1%)	40 (69.0%)	7 (12.1%)	1 + 2 (1), 1 + 3 (4), 2 + 3 (2)

Genotypes were defined based on single nucleotide polymorphisms at positions 85 and 248 of the mitochondrial large subunit rRNA (mtLSUrRNA) gene (Beard et al., 2000). Mixed infections indicate the presence of more than one genotype in the same sample, with subtypes specified in the last column. Percentages are calculated relative to the total number of samples within each group.

## Discussion

Advances in molecular methods have substantially improved the characterization of *Pneumocystis jirovecii* in clinical populations, providing important insights into strain diversity and epidemiology. In this study, we successfully sequenced 58 PCR-positive respiratory samples obtained from distinct immunocompromised populations attending a large tertiary-care hospital complex and identified three mtLSUrRNA genotypes, including cases of mixed infection.

In our cohort, genotype 3 predominated across all clinical groups, contrasting with Brazilian data from Pederiva et al. (2012), who reported a higher prevalence of genotype 2 (Pederiva et al, 2012), but consistent with findings from Africa (Miller et al., 2003; Hauser et al., 1997). Beard et al. (2000) described a predominance of genotypes 1 and 2 in U.S. isolates (Beard et al., 2000), reinforcing the influence of geographic and host-related factors on genotype distribution. The predominance of genotype 3 observed in this study may reflect underlying regional genotype distribution rather than shared exposure or institutional circulation, and should therefore be interpreted with caution in the absence of detailed epidemiological data. Mixed infections were identified in 12% of cases, comparable to rates reported in Spain (3.7%), northern India (6%), and the United States (>10%) (Hauser et al., 1997; Beard et al., 2000; Hauser et al., 2001), further supporting the genetic diversity of *P. jirovecii*. The coexistence of multiple genotypes may reflect exposure to distinct circulating strains and raises important questions regarding both shared exposure in high-complexity healthcare environments and host-pathogen interactions. Co-infection with multiple genotypes could influence disease severity, immune response, and therapeutic outcomes, although current evidence remains limited. In addition, the presence of different strains within the same host may facilitate genetic recombination, potentially contributing to variations in virulence or antifungal susceptibility. This is particularly relevant in immunosuppressed populations, including transplant recipients and patients with hematologic malignancies, where nosocomial outbreaks of PCP have been documented (Yiannakis and Boswell, 2016). Further studies integrating molecular and clinical data are needed to clarify the clinical significance of mixed infections in P. jirovecii.

Stratification of patients according to underlying disease revealed that genotype 3 was the most common among HIV-positive individuals, followed by genotype 2. These findings are consistent with those of Jarboui et al., who also analyzed genotype distribution by underlying disease in different geographic regions (Jarboui et al., 2013). The mtLSUrRNA gene is widely used for the detection of *P. jirovecii*, as it represents a stable and reliable locus for genotypic characterization (Wakefield et al., 1990; Beard et al., 2000).

This study has limitations, including its single-center design and relatively small sample size, which may limit generalizability. In addition, not all PCR-positive samples were successfully sequenced, likely due to variability in DNA quantity and quality. Although this may reflect differences in fungal burden, a formal comparison between sequenced and non-sequenced samples was not performed, representing a potential source of selection bias. As a study based on a single genetic locus (mtLSUrRNA), our analysis has limited discriminatory resolution. Although this marker is widely used and provides a stable framework for describing genotype distribution and detecting mixed infections, it does not allow precise strain differentiation or definitive assessment of transmission pathways. Nevertheless, this study represents one of the first genotyping investigations of *P. jirovecii* in a large tertiary-care hospital in Latin America and highlights the need for future studies using higher-resolution approaches, such as multilocus sequence typing (MLST), to better characterize strain diversity, transmission patterns, and potential associations with clinical outcomes and antifungal resistance. As a preliminary report demonstrating the predominance of genotype 3 across patient groups, further analyses integrating clinical and epidemiological data are required to better define acquisition dynamics and potential shared exposure. The observed genotype distribution may be influenced by multiple factors, including regional background prevalence, potential sampling bias, heterogeneity of the study population, and the limited discriminatory resolution of single-locus typing. These factors should be considered when interpreting the findings and may limit inferences regarding transmission dynamics. In addition, detailed clinical and epidemiological data were not available, including information on hospitalization timing, ward distribution, potential temporal clustering, prophylaxis use, and clinical characterization of infection versus colonization. The absence of these data limits the interpretation of genotype patterns and precludes assessment of transmission dynamics. The clinical implications of mixed infections remain uncertain but may involve differences in pathogenicity, host-pathogen interactions, therapeutic response, or disease severity, and could facilitate recombination between strains with altered virulence or drug susceptibility. Although DHPS polymorphisms associated with sulfa resistance were not evaluated, the detection of multiple genotypes highlights the importance of integrating molecular typing with antifungal resistance profiling. The potential for transmission among immunosuppressed patients warrants further investigation to inform nosocomial infection control strategies; however, formal policies cannot be defined based on the present data. Although the samples were collected between 2014 and 2015, the predominance of genotype 3 remains a valid finding for that period. In the context of limited regional data and relatively stable genotype patterns, these results serve as a reference for future comparative and longitudinal studies.

In this descriptive molecular epidemiology study, genotype 3 predominated across diverse immunocompromised populations within a large tertiary-care hospital. These findings should be interpreted as hypothesis-generating and may reflect underlying genotype distribution or shared environmental exposure rather than direct transmission. Further studies incorporating higher-resolution typing methods and clinical correlation are needed to better characterize strain diversity and potential transmission pathways.

## Data Availability

The original contributions presented in the study are included in the article/supplementary material, further inquiries can be directed to the corresponding author/s.
